# Beyond recognition: gendered violence and the critique of political economy in Croatia

**DOI:** 10.3389/fsoc.2025.1656897

**Published:** 2025-09-24

**Authors:** Jana Kujundžić

**Affiliations:** Department of Social Sciences, Northumbria University, Newcastle Upon Tyne, United Kingdom

**Keywords:** gendered violence, Croatia, Yugoslavia, political economy, social reproduction, redistribution

## Abstract

This paper critiques the understanding of gendered violence solely through the lens of recognition politics without addressing the politics of redistribution in post-conflict, post-socialist Croatia. Since the 1990s, privatization and transition period have eroded social security nets and workers' rights, even as legal reforms and international conventions on victims' protections were incorporated into the Croatian legal system, mainly by EU-funded civil society projects. Persistent underfunding of health, education and social welfare systems undermines meaningful efforts to tackle gendered violence. Drawing on in-depth expert interviews with members of the judiciary, police, social welfare organizations, feminist NGOs, and women's shelters, the paper highlights issues within the legal and social welfare systems through a Marxist-feminist lens. Survivors face the imperative to engage in precarious work, incur significant costs when engaging the legal system, and struggle with limited access to welfare and care services. At the same time, the privileged status of war veterans reinforces social hierarchies and intensifies gendered inequalities. These findings suggest that combating gendered violence cannot be separated from struggles against capitalist exploitation. By connecting recognition and redistribution politics, the paper situates gendered violence within the erosion of post-socialist welfare systems and the persistence of militarized privilege.

## 1 Introduction

This article will emphasize the need for redistribution as essential in fighting gendered violence. The imperative to work as a way to escape violence, the costs of engaging with the legal system and the prevailing significance of war veterans and their material and symbolic privileges in the Croatian society exemplify the inadequacy of mere recognition of gendered violence as a societal issue in capitalism. Rather than treating Marxist feminism, gender-based violence (GBV) studies and scholarship on post-conflict Croatia as separate and demarcated fields, this paper brings them into critical dialogue. It argues that any meaningful understanding of gendered violence in post-conflict and post-socialist societies needs to address the interplay between militarism, growth-oriented economy and social reproduction as an approach which is underdeveloped in current literature. Most studies of GBV in Croatia have focused on institutional reforms, victims' rights, and the role of civil society in aligning the domestic legal framework with international standards. While valuable, this emphasis on recognition politics overlooks the material conditions that shape both vulnerability to violence and access to support networks. This paper addresses this gap by drawing on Marxist feminism to analyze how privatization, welfare retrenchment and labor precarity, key features of Croatia's post-war transition, constrain the possibilities of survivors of violence to access meaningful support, care and protection.

Through empirical analysis of expert interviews with members of the judiciary, police, social welfare organizations, feminist NGOs, and women's shelters, this paper explores three interconnected themes: the imperative of work for survivors in capitalism, the economic and bureaucratic cost of engaging with the legal system, and the privileged status of war veterans in shaping gendered hierarchies of recognition and harm. In doing so, it offers a conceptual contribution to both Marxist feminist theory and critical GBV scholarship by situating gendered violence within the broader context of post-conflict and post-socialist restructuring and its everyday consequences.

This paper draws on a set of key concepts to frame its analysis of gendered violence in post-socialist Croatia, including gendered violence, political economy, social reproduction and redistribution. Gendered violence is understood as a complex phenomenon, as defined by Duff and Jenkins (2021), referring to violence that is rooted in and reinforces hierarchical norms of gender, targeting specific groups of people such as women, girls and gender non-conforming people, based on their actual or perceived deviation from socially constructed, heteronormative ideas of masculinity and femininity. Political economy is broadly understood as the study and analysis of the interrelationship of the economy, politics and society. Social reproduction can be defined as a concept that encapsulates life-making activities such as biological reproduction, household care, labor reproduction and community building (Elias and Rai, 2019; Arruzza and Gawel, 2020; Munro, 2021). Redistribution refers to reducing inequality of the exploited class by demanding a more just distribution of wealth (Fraser, 2013).

In Croatia, the socio-economic and political transition from socialism to capitalism affected women in numerous ways. Most notably, it resulted in a high proliferation of low-paid, temporary jobs and long periods of unemployment, while simultaneously encouraging women into idealized roles of homemakers and mothers by the nation-state-building project, coupled with the support of the Catholic Church (Žarkov, 2003; Galić, 2018). During the EU accession process before 2013, Croatia had to consolidate its legislation with the EU legal *acquis*, subsequently adopting many directives and regulations related to gendered violence and victim protection (Radačić, 2014), while simultaneously liberalizing and privatizing public services. After the EU accession in 2013, there has been evidence of democratic backsliding in the areas of the judiciary, independent media and national-level governance (Čepo, 2020). While the concept of “illiberalism” has been popularized as a critique of populist right-wing governments and democratic backsliding in Central and Eastern Europe (Kovats, 2017; Korolczuk and Graff, 2018; Čepo, 2020), it neglects the problems and violence inherent in the liberal nation-states. Liberal democracies are implicated in the imperial, colonial and racialized capitalist order that often manifests in repressive and violent forms of state control (Lottholz, 2024). One of these violent and repressive forms can be observed in the state's repression of protests that threaten the property-order of liberal democracies (Duff, 2017). In other words, the idea of (human) rights is often in conflict with the sanctity and protection of private property, in which the latter takes precedence. In connection with gendered violence, these repressive forms are also observable in state interventions and policing, which can further harm and traumatize survivors of gendered violence, especially those from marginalized groups (Miller, 2001; Goodmark, 2023; Cowan, 2024). Repression and violence can also be observed in state institutions tasked with reproduction, which enforce and implement the state's rules (Munro, 2021). In other words, health, education and social welfare services can perform policing functions, further harming the survivors of violence by denying services, engaging in needs-interpretation and gatekeeping. Brown (1995, p. 169) frames this even more straightforwardly: “Whether one is dealing with the state, the Mafia, parents, pimps, police, or husbands, the heavy price of institutionalized protection is always a measure of dependence and agreement to abide by the protector's rule.”

Furthermore, Croatia, as the latest country to join the EU, is often portrayed as a success story of the enlargement process, which overlooks the various socio-economic issues that have arisen since accession. Čepo's (2020) analysis of democratic backsliding focuses on the corruption and control of state assets by the Croatian Democratic Union (HDZ), which won the first multiparty elections in Croatia in 1990 and is currently in power. HDZ is also the first political party to be convicted of corruption^1^ by the Supreme Court of Croatia (Čepo, 2020). However, a narrow focus on corruption and crony capitalism, which are critiques often leveled at post-socialist economies, sidelines the role of the EU's neocolonial free-market ideology that mandated privatization and fiscalization. As Stubbs and Žitko (2024, p. 6) argue, by 2016, “The Croatian economy had already morphed into a deindustrialized service economy integrated into the EU landscape of institutionalized austerity and surveillance with low fiscal capacity cushioned only by increased dependence on EU cohesion funds.” The mass industrialization led by revolutionary Yugoslav leader Josip Broz Tito after the Second World War prompted a large number of young women to find jobs in the textile sector (Bonfiglioli, 2019). After the Yugoslav war in the 1990s, many factories closed, resulting in liquidation, cutbacks and constraining thousands of textile workers into dubious and exploitative private-sector occupations. Conditions in which gendered violence abounds are linked to the effects of deindustrialization, specifically economic deprivation and community insecurity (Goodmark, 2018). To sum up, the democratic backsliding and deindustrialization have made things worse for women in Croatia, especially in relation to gendered violence.

Moreover, the failed promise of prosperity in the so-called free market is evident in Croatia's dependent market economy, as evidenced by the foreign trade balance that has been in deficit almost continuously since the dissolution of Yugoslavia in the 1990s (Gužvica and Bilić, 2024). Croatia consistently imports more than it exports, and this deficit is usually measured in billions of dollars, which is one of the most reliable indicators of a country's dependent market economy (Gužvica and Bilić, 2024). The transition period, privatization and different stages of Croatia's capitalist development were far more complex than explained above; however, in connection to gendered violence, this is evident in the weakened state services and the shifting burden of social reproduction onto the individuals or the market. Bhattacharya (2013) explained that capitalism benefits from unpaid labor in terms of social reproduction and minimal social wage investment. This dependence of production on social reproduction is crucial for understanding gendered violence as an integral part of the political economy of gendered relations (Bhattacharya, 2013). The lack of social security nets and the precarious position of working-class women victims of violence in Croatia complicates the picture of combating gendered violence through legislation and NGO projects.

The imperative placed by institutions on victims of abuse to leave their abusers sidelines the fact that the divorce procedure has the power to determine the materialist conditions of women's lives, especially when they are also involved in the criminal procedure as victims of gendered violence. Divorce might leave these women financially destitute, and their reports of abuse might be used against them. Following the process of privatization and de-institutionalization, the underfunded and understaffed social welfare sector (Stubbs and Sertić, 1996; Stubbs and Lendvai-Bainton, 2020) relies on the few NGOs that provide social services (Bežovan, 2003). Both the social welfare and NGO sectors struggle to prevent cases of gendered violence due to the contradictory nature of their reproductive work, which also serves the repressive function of the capitalist state by disciplining their working-class “clients” into workers (Munro, 2021).

This article is structured as follows: the section below presents the theoretical framework, starting with the literature review of relevant Marxist-feminist literature that helps to situate the issue of gendered violence in materialist terms. Following this, an overview of the literature closely related to the specific post-Yugoslav context of women's labor rights and gendered violence is provided. After that, the methodology section explains the overarching framework, data collection and analysis process and ethical considerations. The discussion and findings section focuses on three themes: the first, the critique of the imperative of work for survivors of gendered violence. Second, the focus on costs of reporting abuse, including the issue of access to services and facilities. Third, the critique of the special status of war veterans is related to gendered violence in both symbolic and material terms. Finally, I conclude by arguing that combating gendered violence is inextricably linked to the broader struggle against exploitation in capitalism.

## 2 The importance of the Marxist-feminist lens on gendered violence

Economic crises since the 2008 crash, austerity, the pandemic, geopolitical conflicts and climate catastrophes have led to a deepening of economic inequality, influencing critiques of postmodernism, postfeminism, and intersectional approaches, which often overlook the totality of capitalist social relations. Much of feminist theory in the past two decades has tended toward a poststructuralist understanding of gendered violence, but has failed to historicize gendered violence and unearth its material underpinnings. As Bhattacharya (2013) argued, to combat gendered violence and inequality, Marxist feminists^2^ need to investigate the material basis of gender relations under capitalism, especially how neoliberalism has reorganized social reproduction.

Furthermore, this article will avoid reducing the problem of patriarchy and gendered violence to individual antagonisms between men and women. This reductionism is an inherent feature of liberal feminism, which holds onto an ahistorical understanding of patriarchy and decontextualized considerations of the marginalization and discrimination experienced by women (Burcar, 2020). To understand gendered violence, which is a product of the political economy of gender relations, the contradictory nature of the dependence of production on social reproduction needs to be problematized (Bhattacharya, 2013). Social reproduction theory (SRT) links production and reproduction, showing how capitalism depends on both wage labor and unpaid domestic labor while benefiting from unpaid reproductive labor (Bhattacharya, 2017). Moreover, the burden on families, especially women, is increased by the privatization and individualization of unpaid labor in the home, public services funded by the state, and paid services provided by the market.

SRT seeks to demonstrate how various oppressions are constitutive of labor relations rather than solely focusing on the gendered dimension of social reproduction as assumed in what can be called reductive feminisms. From a Marxist viewpoint, class is qualitatively different from race and gender and cannot be reduced to just another form of oppression (Gimenez, 2001). The underpinning, unnamed force that affects all interactions related to class, race and gender is the class power itself. The liberal critique of what is deemed as “class reductionism” led to gender, race and ethnic reductionisms (Kandal, 1995; Gimenez, 2001). In other words, the misunderstanding of class resulted in purely semantic struggles, with the endless reproduction of terms that describe the relationship between gender, race and class, defanged from the potential for revolutionary class struggle. Furthermore, understanding oppression as manufactured and reinforced by social relations through a dialectical model is more beneficial than taxonomies of oppression for building non-identitarian solidarity (Lewis, 2016).

SRT emphasizes the work that creates and renews life, showing that the possibility for profit-making work depends on life-making work. In other words, the work predominantly done by women is a prerequisite for sustaining the daily lives of laborers. This work is not only done at home but is also provided by the state through social provisioning, which largely comprises feminized professions such as teaching, nursing and social work. SRT's analysis also helps to show how violence is closely related to work, because violence is constitutive of work in capitalism through coercion and restrictions. Furthermore, as mentioned above, Munro (2021) argued it is not possible to separate state social welfare provisions from their repressive and violent functions, as evidenced in the contradictory role of teachers, nurses and social workers who are tasked with providing services but at the same time disciplining and repressing the working class.

There have been increasing critiques from the Left on the futility of identity politics, which has deviated from its original radical meaning (Drucker, 2015; Lewis, 2016; Noyé and Rebucini, 2021; Táíwò, 2022; Sarkar, 2025). The term was originally coined by the Combahee River Collective, whose members believed in internationalism and the struggle against the US-led West's colonial, imperialist, and capitalist domination (Combahee River Collective, 2017). The Combahee River Collective comprised women veterans of the Black Panther Party and other antiracist organizations. As Black feminists, they have established a tradition that explicitly rejected individualizing and separating racism from sexism and vice versa. Furthermore, they understood racism and poverty as interlinked in the capitalist society, and necessarily rejected middle-class strategies for women's liberation, which ignored the centrality of class in working-class and poor women's lives. Instead, they argued for a redistribution of resources based on the collective needs of the most oppressed.

Drawing on the need to redistribute resources, Fraser (2013) proposed an understanding of gender in a two-dimensional way: rooted in society's economic structure and status order. Or in other words, it is concerned with both recognition and redistribution. This helps us imagine gendered justice as constitutive of both changing the economic structure that creates the gendered division of labor, and the status order of contemporary society which institutionalizes androcentric values (Fraser, 2013). To clarify, the issue of misrecognition is related to women's subordinate status in society, where they are not considered equal to men in all areas of social life. Fraser (2013) proposes a non-identitarian politics of recognition in order to avoid colluding with neoliberalism. The gendered subordination has long been an explanation for gendered violence by radical feminists (Duff, 2018). However, overcoming gendered subordination necessarily requires the redistribution of resources to avoid mere symbolism of recognition. For example, new forms of work, such as part-time work, work-from-home arrangements, and additional work in private or family businesses, were constructed by the capitalist order in Croatia as family and mother-friendly employment (Burcar, 2020). These policies were introduced by recognition of the unique status of mothers and motherhood in relation to labor. However, these forms of work have highly negative consequences for the socio-economic status of women as well as segregation effects. They also enable the deinstitutionalization of public care networks and facilitate the exploitation of cheap labor power. This is in vast contrast to the previous socialist labor policies of Yugoslavia, which guaranteed women's rights to permanent and full-time employment alongside newly established supportive public services such as kindergartens and nurseries (Burcar, 2020). Here we can see how capitalism's feminization of poverty reorganizes social reproduction and pushes women into the idealized roles of homemakers. Naturalization of gendered roles, isolation and economic dependence increase vulnerability to gendered violence.

Yugoslavia's self-management workplace model was a unique phenomenon nestled between US capitalism and Soviet communism. Based on changes in ownership of the means of production, women in Yugoslavia were granted equal rights across all areas of state, economic and socio-political life. According to the Yugoslav Constitution of 1946, women were not only given the right to vote but also the right to equal pay, a right that the West introduced only in the 1970s (Burcar, 2020). This marked just the beginning of the deliberate process of socioeconomic emancipation for women, whose success depended on structural changes to the political and economic systems, intertwined with the regulation of relations and division of labor. In other words, it wasn't merely legislative changes that aided the socioeconomic emancipation of women, but the transformation of the social order and political and economic system toward socialism. Socialist policies promoted women's full employment while supporting them through newly established public services, marking a progressive break with the previous historical period of the Kingdom of Yugoslavia (1929–1945), during which women lacked any^3^ political or property rights (Burcar, 2020). This period was also shaped by the participation of working-class and peasant women in workers' antifascist and communist organizations (Kocman, 1978). Nevertheless, for the first time in history after 1945, women across Yugoslavia obtained legal protection and rights based on secular state principles. These included equal rights to men in inheritance and family law (particularly the right to request divorce), political rights (such as voting rights), the right to work and equal pay, the right to abortion and access to social protection (Gudac Dodic, 2006). The right to abortion was enshrined in 1974 as “the right to family planning” in a Constitutional provision (Gudac Dodic, 2006). The Yugoslav abortion law was considered revolutionary at that time, as abortion was still criminalized in many other European countries (Gudac Dodic, 2006).

The Antifascist Women's Front (AFŽ), formed in 1942, played a key role in mobilizing women partisans who fought alongside men against fascist and Nazi occupation, and many of its members later became leaders of partisans' boards (Jovanovic, 2015). AFŽ was also responsible for, among other activities, a nationwide literacy campaign that taught women how to read, write and be politically active in their local communities (Zaviršek, 2008). AFŽ was disbanded in 1953, which liberal feminists saw as a defeat for women's rights and a return to institutionalized patriarchy, even though emancipatory politics continued to be systematically embedded in many sessions of the Yugoslav leadership of the socialist alliance and influenced the discussions held by the UN under the banner of “women's issues” (Burcar, 2020; Bonfiglioli, 2021). Furthermore, the liberal feminist push for separate women's organizations and lobbies is rooted in identity politics divorced from socio-economic systems of exploitation and material conditions. As Burcar 2020) argued, citing Vida Tomšić (1913-1998), a prominent Yugoslav partisan and politician who played a vital role in both the Non-Aligned Movement and UN settings,^4^ overcoming women's subordination is unattainable within isolated and segregated women's organizations because it is a societal and structural issue that requires changes in material conditions and a comprehensive overhaul of society as a whole.

Another common liberal and radical feminist critique of socialist Yugoslavia is that Yugoslavia failed to eradicate patriarchal institutions, and that gendered violence remained widespread and unaddressed (Ramet, 1999; Lóránd, 2019). The issue with this argument is that addressing gendered violence is seen solely through a lens of legislative and carceral responses of the state, rather than through structural socio-economic changes that enable not only the economic independence of individual women but also a collectivization of care.

The perceived shortcomings of women's rights within socialism led to the formation of a network of women's groups in the 1970s, referring to themselves as the new Yugoslav feminists (Lóránd, 2019). They were inspired by what is commonly known as the “second wave” of feminism in the 1960s and 1970s in the US and Western Europe. While the wave analogy helps to illustrate the fragmentation of feminist activism, it is an oversimplification (Olufemi, 2020). Despite the complexity of the so-called second wave, problematic assumptions and legacies continue from Western radical feminist literature of that period. Essentialist approaches to gender difference, the positioning of all women as victims, equating (heterosexual) sex with violence, neglecting race and class issues, whitewashing, heteronormativity and Western hegemony are some of the problems of second wave feminism criticized by Marxist and intersectional feminists (Rodriguez Martinez, 2011). I argue that some of these issues were uncritically transferred to the Yugoslav context of the 1970s, which had already experienced liberalization, student protests, nationalist sentiments, and an opening to the West (Haug, 2012; Lóránd, 2019). Instead of turning to revolutionary women's writings from non-aligned countries of the Global South, the Yugoslav new feminists were drawn to Western radical feminists such as Dobash and Dobash, Brownmiller, Thompson and Temkin (Lóránd, 2019), whose arguments were used to advocate for state protection against gendered violence and carceral systems solutions. These approaches to gendered violence influenced feminist activism after the dissolution of Yugoslavia in the 1990s by emphasizing gender oppression as a fundamental cause of gender inequality, ignoring the class-based exploitation.

Rooted in this focus on domestic violence within Western radical feminist literature, the first SOS Phoneline was established in 1988 in Zagreb, followed by SOS Phonelines in Ljubljana in 1989 and Belgrade in 1990. The war in the 1990s had a damaging impact on the women's network in former Yugoslavia, with some activists aligning with an ethno-nationalistic agenda (Miškovska Kajevska, 2017). Those feminists who emphasized anti-nationalism, transnational solidarity and peace were publicly denounced as traitors of their homeland (Boric, 2003). After the war, feminism in the former Yugoslavia essentially became subsumed under professionalization in NGOs funded by local and international funding bodies (Jansen, 2005).

## 3 The political and economic consequences of wartime and transition to capitalism

In 1994, UNICEF found that domestic abuse was widespread during the so-called transition^5^ period in Croatia (UNICEF, 1994). Despite democratic reforms in the 1990s, women's roles in the public diminished, and many faced unemployment. Although there are no empirical studies in Croatia to confirm the idea that the war exacerbated domestic violence, it had devastating consequences on the position of women more generally (UNICEF, 1994). Many women became widows struggling to adapt to their new family role, whilst some men came back from the war with PTSD. The war also increased societal tolerance for violence, and the transition period was marked by decreased social security and rises in unemployment and poverty. Between 1995 and 2002, there was an increase in police interventions for domestic violence (UNICEF, 1994). Data from the Women's Counseling Center for the period from 1997 to 1999 also showed that 57% of women sought help due to domestic violence (Ajduković and Pavleković, 2003).

The consequences of the transition period in the 1990s were not unique to Croatia but had similar effects across the region. Research conducted in 1999 and 2000 in North Macedonia, Serbia, Hungary and Bulgaria discovered that 53.6% of women thought that the quality of their family life had worsened in the past 10 years (Nikolić-Ristanović and Dokmanović, 2006). These countries had the most significant decline in the number of employed people between 1989 and 1997 (Nikolić-Ristanović, 2002). Job losses strained family relationships, leading to worsened financial situations. On the other hand, the sudden acquisition of wealth for certain members of society^6^ also contributed to the deterioration of relationships, especially if the husband entered the *nouveau riche* echelon (Nikolić-Ristanović and Dokmanović, 2006). The attainment of a better social and economic position for these men entrenched notions of their superior masculinity and the renewal of traditionalism regarding gender roles in the family. In contrast, the majority of men in the region of former Yugoslavia have seen their social and economic position deteriorate after the war and during the transition to capitalism, contributing to the social stress and isolation, which increased the risk of domestic violence (Nikolić-Ristanović and Dokmanović, 2006).

An additional risk factor was the so-called crisis of femininity from the socialist period, related to women's job losses (Nikolić-Ristanović, 2002). For well-educated, middle-class women who had enjoyed relative economic independence during communism, this meant feeling degraded in their new role as homemakers. For working-class women, the consequences were more dire, as they lost material security, collective empowerment and the symbolic valorization of their work in textile factories (Bonfiglioli, 2020). Factors connected with the transition period also included distrust in state institutions and corruption (Nikolić-Ristanović and Dokmanović, 2006). The increased influence of the Catholic Church in Croatia marked the transition period, coupled with the ethno-nationalistic agenda, which facilitated the reorganization of social reproduction, isolating women in the nuclear families.

The discourse regarding women in Yugoslavia dramatically shifted as a result of the war in the 1990s, from emancipated, partisan, politically and socially engaged Yugoslav women to ethnically marked (mostly Croatian and Bosnian) victims of wartime sexual violence (Žarkov and Drezgić, 2006). State-controlled media in Croatia essentially exploited wartime rape to further the political agenda of the nation-state building project (Zarkov, 2001). Wartime sexual violence was also utilized by international feminist scholarship in insisting on ethnic markers of victims and perpetrators (Kesic, 1994; Olick and Stiglmayer, 1995; Žarkov, 2007). The focus on ethnic dimensions colored by nationalistic sentiments still prevails in the post-war period, as evidenced by the implementation of the Law on the Rights of Victims of Sexual Violence in the Homeland War^7^ under the purview of the Ministry of War Veterans.^8^ There were complaints of the Ministry‘s biases, highlighted in a refusal of Serbian victim claim of wartime rape by the Croatian Armed Forces in 1993 (Nacional, 2018).

Economic changes following the transition to a market economy had significant consequences for collective life, mediated by the war, privatization, and deindustrialization. Growth as the primary driver in capitalist economies meant reducing the state provisions and services. Consequently, private companies, civil organizations and individuals were encouraged to take responsibility for services that the state no longer provides (Goodmark, 2018). In other words, while legislation and legal procedures on domestic and sexual violence were introduced in the Croatian legislation, workers' rights and protections were curtailed, the commons were privatized, and the burden of care and welfare individualized. Macroeconomic politics, deindustrialization and neoliberal economic policies are all linked to gendered violence (Goodmark, 2018). Women were the first to be laid off during the transition period and “went back” to their traditional roles of homemakers (Kamenov and Galic, 2011). The employment opportunities became scarcer, especially for women with lower qualifications who had more than one child and were in older age groups (Kamenov and Galic, 2011). The proliferation of low-wage, part-time and precarious employment for these women meant that they were at greater risk of becoming economically dependent on their husbands and partners, complicating their decision to leave violent relationships.

## 4 Methodology

This paper draws on data from a larger research project examining the political and legal dimensions of gendered violence in Croatia based on in-depth expert interviews. The interviewees included members of the judiciary, police, social welfare, feminist NGOs, women's shelters, journalists, and academics. Thematic analysis was used to derive broad themes from the coding nodes after categorizing and analytically reflecting upon them by examining concepts and meanings (Saldaña, 2016). The topic of this article corresponds to the delineated theme named initially “The Political Economy of Gender-Based Violence”.

In-depth interviews were selected as the most appropriate method to explore the cases of GBV as explained by the experts in their own words and through their lived experiences. While court data and case reports could have provided information on GBV cases, the expressive subjective narratives (Arsel, 2017) of the experts went beyond simple descriptions and case analysis, incorporating broader socio-political and economic realities of daily life. The inherent power imbalance in the interview process can be addressed by adopting a more collaborative style of interviewing. In other words, the interviews were not meant to be interrogations but rather a conversation aimed at building knowledge collectively (Kvale and Brinkmann, 2009). Interviewing facilitated interaction and knowledge (co)production between the researcher and participants, as well as direct witnessing of real-life challenges. For example, reading about social workers' understaffing and underfunding in a report (Rajter, 2015) was different from observing the dilapidated building, an elderly security guard with mobility issues and several towers of paper case files in a cramped office, that had to be rearranged by the social worker to facilitate the interview. Furthermore, due to the sensitivity of the topic, the interview as a method allowed for the expression of a wide array of emotions during the research. The emotions experienced during this process can be valuable in interpreting data and critically reflecting on the research process (Ryan-Flood and Gill, 2010). For instance, the frustrations, anger, and sadness expressed by some participants regarding the failings of the welfare and legal systems to assist or protect survivors facilitated “affective solidarity”, which is based not on shared identity or empathy, but on the desire to bring about social change (Hemmings, 2012). In other words, the presence and experience of discomfort and unease with dominant norms and power relations foster a desire for societal transformation. Understanding the broader injustice within the legal and welfare systems creates a space for feminist and materialist politics of change.

Participants were selected through established professional and activist networks, focusing on experts with experience related to gendered violence. This experience included working directly with survivors, policy-making, research or clinical practice. For this research, non-probability purposive sampling was deemed the most appropriate method. The main selection criterion was professional experience with gendered violence, regardless of specific demographic characteristics. Data collection took place from 2018 to 2019 and was conducted in person in Croatia. All interviews lasted approximately 45–90 min. Twenty-five in-depth interviews were carried out, audio recorded, and transcribed verbatim by hand. Only selected quotes were translated from Croatian to English. Ethical approval was granted by the University of Essex. Participants received an information sheet and a consent form, ensuring their anonymity. Due to the limited pool of Croatian experts working on gendered violence issues, my responsibility as a researcher was to protect the privacy and anonymity of participants, ensuring that nothing said could subsequently harm them personally or professionally. Each participant was assigned a pseudonym chosen randomly from an online database of common Croatian surnames. Guided by the practical instructions for researchers and activists addressing violence against women, the wellbeing of participants remained central to the research process (Ellsberg et al., 2005). This involved making participants aware of their right to withdraw at any stage of the interview and employing strategies such as active listening, empathizing, and reflecting throughout the conversations.

The interview guide was semi-structured, based on broadly identified topics, including concrete examples of professional encounters with cases of gendered violence, views on socialization, gendered roles, the socio-economic context in relation to gendered violence, and opinions and involvement in policy-making and law-drafting groups. Thematic analysis was used to interpret the data, categorizing and making sense of it (Naeem et al., 2023). After the initial analysis, additional themes were identified. The coding process employed a hybrid approach, combining manual methods with computer-assisted qualitative data analysis software, NVivo. Quotes from interview participants included in this paper were carefully selected from in-depth interviews, which were transcribed into dozens of pages of text. The quotes chosen support the presented interpretations and explanations, primarily illustrating how the findings and insights emerged from the data collected. The main purpose of including direct quotes from the interviews is to demonstrate how the findings and interpretations derive from the data (Patton, 2015). In this way, experts' views are presented in their own words. Only relevant quotes have been included and edited for clarity, given the limited scope of the paper.

As described elsewhere (Kujundžić, 2022), my own positionality as a researcher involved a certain level of secrecy and silence, which are inherent to every research process (Ryan-Flood and Gill, 2010). I used strategic silence to protect the data collection process by not revealing my political positions, aiming to avoid antagonizing or provoking some of the participants. Both Marxism and feminism have rich and diverse theoretical foundations, and neither term is immediately apparent or transparent in public discourse in Croatia. As a former NGO worker, volunteer, and activist in various progressive movements, I had to navigate the tensions between these roles and my role as a researcher. These tensions were eased in the company of some participants who were also actively involved in or supported activism around the issue of GBV. Moreover, the success of the research partly depends on the researcher's experiential knowledge of the phenomenon (Fook, 1999). In other words, both my previous work and activist experience supported me during the data collection process and influenced the data analysis. As previously mentioned, the affective solidarity (Hemmings, 2012) is developed from feminist reflexivity, which arises from an experience of dissonance between the self and social expectations, leading to reflexive politicization.

## 5 Findings and discussion

Three core themes were identified in the participants' narratives related to gendered violence and the political economy, which will be explored in the sections below. The first theme will demonstrate how the meaning of work has changed from socialism to capitalism and the implications of that for survivors of gendered violence. The second exemplifies the costs of reporting the abuse as well as issues of accessing survivor-oriented services and facilities. The third explores the symbolic and material status of war veterans in the Croatian society and its direct implications on gendered violence. All themes reveal the complicated picture of fighting gendered violence in capitalism, where the focus is on recognition rather than redistribution.

a. “Without a job, there is no escape out of violence”- The Imperative of Work for Survivors of Gendered Violence.

The global economic crisis of 2008, arriving only 12 years after the end of the war in Croatia, further disadvantaged women and increased their financial and labor inequality vis-à-vis men. Compared to men, women, on average, pay a higher price for negative economic trends (Goodmark, 2018). The socio-economic circumstances of the economic crisis further exacerbated economic violence. While the broader economic context is occasionally acknowledged in literature on economic violence, it is often conceptualized as a special form of domestic violence against women, including psychological, physical, or sexual abuse (Stylianou, 2018; Postmus et al., 2020).

Croatian Law on Protection from Domestic Violence defines economic violence as “prohibiting or debilitating the use of personal or joint property, disposing of personal income or property acquired through personal work or inheritance, preventing employment, denying funds to maintain a joint household and caring for children” (Official Gazette of the Republic of Croatia 126/19). Economic violence is often connected to other forms of domestic abuse in cases where the victim does not have sufficient funds or an independent income to facilitate their escape from the relationship. Having to navigate the legal system as a result of reporting gendered violence often requires financial support, even if the state provides free legal aid. Attending court dates might mean missing time from work and incurring costs for transportation and childcare.

Mandić, a former prosecutor and a private practice lawyer,^9^ told me she had a recent divorce case in which the woman did not know anything about her and her husband's finances.

I asked her: You have been working for 38 years, how much was your salary and where did that money go? She told me: ‘I don't know, I would give him [the husband] my whole salary every month'. She knew nothing about the bills, about the mortgage, or even which bank he had his account with.

At first glance, the case above illustrates a patriarchal arrangement in which the husband, as the head of the family, oversees all the finances, leaving the wife in a desolate position if she wishes to leave. However, it also points to the hidden assumption of individual financial responsibility rather than a collective one in which the society as a whole takes care of its members. There is a tendency in current gendered violence advocacy in Croatia to stress the importance of employment as a way out of an abusive relationship. The argument goes that an unemployed, economically dependent woman has significantly reduced opportunities to confront violence and achieve successful parenting.

For example, the Autonomous Women's House Zagreb, in their study on economic violence in Croatia, discusses the manifest and latent employment functions (Maslić Seršić, 2010). Manifest employment functions can be seen as directly related to income-earning and financial security. In contrast, latent functions are related to time structuring, meeting and socializing with other people, social status and social activity. Unemployment, coupled with abuse, can lead to women becoming socially excluded from society and unable to access support (Maslić Seršić, 2010). However, manifest and latent employment functions, however relevant to the individual, do not seem to address the structural issue with work under capitalism; namely, exploitation, precarity and commodification of time. This lack of acknowledgment also negates the possibility of resistance to capitalist temporality and its emphasis on efficiency, productivity, and segmentation of time in contrast to the commons and community (Sørensen and Wiksell, 2019).

The study goes on to list the violent strategies related to economic abuse such as exclusive control of joint financial resources and deprivation of women's economic autonomy; impoverishing and putting women in a state of financial and material deprivation; placing women in a position of economic dependence on the abuser; violent control and confiscation of personal material resources available to women; forcible obstruction of an employed woman in the performance of her work duties and deprivation of her autonomy in deciding how to manage the financial income (Maslić Seršić, 2010). While the influence of the global economic crisis is acknowledged, the study's recommendations focus on the top-down development of psychosocial and employment programs, as well as the provision of other forms of material and social support. Mutual aid, bolder demands for redistribution, and problematization of the sanctity of private property are not explored in the report.

Research conducted in 2013 focused on economic violence as an often neglected and underreported form of abuse (Sarnavka and Markulin, 2013). The research was conducted in five countries: Croatia, Serbia, Bulgaria, Bosnia and Herzegovina and Montenegro. The main findings were that both employed and unemployed women are exposed to economic violence, although the consequences and forms that it takes are different in these two groups. Only 11.5% of women in Croatia own the property in which they live with their partners (Sarnavka and Markulin, 2013). Most women have raised bank loans during their marriages and partnerships to finance the renovation of apartments or houses in which they lived with their partner's parents, which they repaid alone or have continued to repay them after the relationship or marriage ended (Sarnavka and Markulin, 2013). In already unstable economic conditions, this burden is a significant barrier for women with children who want to leave abusive relationships.

Lawyer Mandić also advanced this point in relation to the scope of her legal work:

I had cases in which all the property was signed in the man's name, and the woman agreed to it and often they took out loans in which she is the co-debtor or the guarantor and if she wants to leave the relationship, he will stop paying the loan and her salary will be repossessed.

Savić, a renowned feminist and the founder of a well-known feminist organization that helps women victims of violence and fights for gender equality,^10^ remarked on the importance of employment and work for women, stemming from the research she conducted on the position of women in Croatia and attitudes toward them:

It was established that there is this tendency in modernity in Croatia, even in the attitudes of the most conservative women, that a woman should work and be employed, even if all their other attitudes are retrograde. They think it is important to get married and give birth but working is important as well. Every third marriage in the urban areas ends up in divorce and then women who did not work fare very badly and often never manage to leave, especially if the relationship was abusive, because they don't have any financial means. Research has also shown that single parents are primarily women and they are at risk, often living on the verge or below the poverty line.

The acceptance of the idea of the working woman can be seen as a legacy of the former socialist system, in which women's emancipation was tied to productive labor. However, the meaning and type of work, self-management of factories, workers' protections and rights have significantly changed since the socialist period. Deindustrialization and privatization mitigated the devaluation of women's industrial work in post-socialist Croatia (Bonfiglioli, 2020). While employment might provide women with the economic opportunity to leave abusive relationships, the proliferation of low-paying, flexible and precarious jobs might further destabilize women's financial situations. The reorganization and privatization of social reproduction also meant that housework in capitalism is presented as an innate women's duty, or work of “love,” and therefore not “real” work which requires adequate financial compensation. At the same time, as competitive labor markets devalue care work done primarily by women, they thrive on women's unpaid labor at home, which enables the social reproduction of workers (Ghodsee, 2018).

Pavlović, a psychologist and a former women's shelter worker,^11^ pointed out that victims should not expect too much help from the system since it is usually the victim's responsibility to secure employment and material conditions:

Without a job there is no escape of violence for any woman. And that is a major issue in Croatia, employment, and employment of women, and especially employment of women victims of abuse who have been outside the job market for a long time. I think every woman who wants to break the cycle of abuse has to primarily think about herself and persist in her decision. No system will do that for her, she needs to do that alone and, in the end, it all falls down on the individual.

Pavlović identifies a systematic problem when it comes to securing financial means for victims of any kind of abuse, including gendered violence. With the deterioration of social security nets and lack of funding for the social welfare sector, including women's shelters and NGOs working with domestic abuse, the sole responsibility for securing adequate financial conditions falls on the individuals. She also elaborated on the issue with state policies designed to employ women victims of domestic violence:

For a time, employers could get incentives for the employment of women victims of violence, and there were subsidies by the state. This was exploited by employers en masse, for some ridiculous salaries of 2000 or 3000 HRK.^12^ But even that starting salary and initial employment meant a lot to [abused] women because a lot of them would enter the labour market for the first time after X years. Some employers would notice them, see that they were valuable and eager to prove themselves, and then hire them on a permanent contract; it was a step out of violence for them. Most women need this financial help because in most of these marriages a woman is unemployed, because if she is employed then there are accusations like: ‘What are you doing, who knows who you're hooking up with,' and when she stops working then he says: ‘You just want to spend my money, you are nobody and nothing without me, you have nothing without me.' Such pressures become so terrifying that she usually resigns herself. And then at work, she says, ‘It's hard for me to be apart from my children', she invents some reason without actually saying what the reason for her resignation is.

As Pavlović explained, the state's incentives can be exploited by employers looking for cheap labor rather than providing opportunities to aid abused women on the path to financial independence. Furthermore, keeping a job while being involved in an abusive relationship is a difficult task. Abused women are less likely to maintain long-term employment and report being late and absent from work than other women (Goodmark, 2018). Abusive partners may deter victims from going to work by destroying job-related documents and work clothes, inflicting visible injuries or harassing them at work. To add insult to injury, state incentives often push women survivors into low-paid and precarious jobs such as carers for the elderly (in Serbo-Croatian: geronto-domaćice). The deinstitutionalisation of elder care has enabled state incentives for both for-profit and non-profit organizations to take on the burden of care that the state no longer provides. EU-funded project in 2017, “Make a Wish -Women's Employment Program”, specifically targeted hard-to-employ women, including survivors of abuse, to care for the elderly in their own homes (Šipuš, 2023). While the project has been deemed successful and beneficial for the local community (Šipuš, 2023), the state has relinquished part of its responsibility for the aging population through the privatization of care and placing the burden of social reproduction on the shoulders of women with the lowest level of employability in the competitive market economy.

b. “The System is Not Working” - Costs of Reporting Abuse.

Getting involved with the legal system requires financial support. Having a legal advocate means not just better representation at court but also insight and guidance through the inner workings of the legal process, which may be confusing or unintelligible for survivors. The presence of a legal advocate may provide a favorable outcome, as it signals the victim's commitment to the legal process, adding legitimacy to her claim, and also adds an additional measure of accountability for state officials (Neumann, 2017). Every victim in a criminal proceeding in Croatia is entitled to a court-mandated attorney, and any attorney can be on the court's list, but this information is not always readily available to victims. In the legislation pre-2011, prosecution for minor bodily injury was undertaken by a private lawsuit filed with the relevant municipal court. This meant that abused women were required to initiate the prosecution themselves by filing a private lawsuit with the relevant municipal court. To do this, the victim must be familiar with her rights and have sufficient financial resources to pay court fees. This can begin at 500 HRK,^13^ rising to 1,800 HRK^14^ to hire an attorney and to pay for a forensic examination, and may further rise to several thousands of HRK to account for attorney expenses and the cost of any court fees on appeal. This changed with the amendments to the Criminal Code in 2011, which dictated that minor bodily injury committed against a spouse, family member or partner is no longer prosecuted privately but officially by the state attorney's office.

Katanić, a private practice lawyer who works for a women's organization,^15^ stated his opinion on the impact of his own presence in the courtroom:

I think the legal aid is necessary, it is so difficult to be alone in the legal process, not only that, but prosecutors and judges look at you differently if you have legal backup and that is clear as day that it goes in favorem to those that have legal backup, and it can harm those who don't.

However, legal representation costs the victim unless it is a state-mandated attorney. According to Katanić, at the time of the interview, the cheapest lawyer would still cost around one thousand HRK plus value-added tax for every legal action in the proceedings.

Survivors of abuse withdraw from proceedings for numerous reasons, including being exposed to exacerbated violence and high financial costs. Even if they stay involved in the proceedings to the end, the final sentence might be imposed as a further financial sanction. Nationwide, judges issue fines in 50.8% of cases of domestic violence (The Advocates for Human Rights, Autonomna Ženska Kuća Zagreb and Bulgarian Gender Research Foundation, 2012). A sentence for the perpetrator of abuse can have financial consequences for the whole family.

Blažević, a retired police officer,^16^ confirmed that most domestic abuse reports are sentenced as misdemeanors, for which the most pronounced sentence is a fine:

If he gets a fine of one thousand five hundred HRK that is punishment for the entire family, which might mean that they cannot buy clothes or books for their children for that month. So, the punishment reflects on the deprivation of the family life, by depriving basic family needs.

Sentencing the perpetrator with a fine may also punish the victim, especially when the victim is economically dependent on the perpetrator or when the payment of the fine must come from joint family funds. There can even be cases where the victim has to pay the perpetrator's fine herself (The Advocates for Human Rights, Autonomna Ženska Kuća Zagreb and Bulgarian Gender Research Foundation, 2012). This type of sentence presents a financial risk that may deter further engagement with the legal system.

Rendulić, a psychologist working at the feminist NGO for sexual rights,^17^ also pointed out that reporting domestic violence often means it will fall under the qualification of a misdemeanor:

The longest prison sentence he can get for domestic abuse is 90 days. After that, he can come home, which in most cases is his own; he is the owner of the property.

Social welfare centers may provide victims with one-off financial assistance for basic expenses, but this is not always an available or sufficient option for victims. This assistance is often not enough for the victim to cover living or legal expenses. Furthermore, some welfare centers do not inform victims of this possibility (The Advocates for Human Rights, Autonomna Ženska Kuća Zagreb and Bulgarian Gender Research Foundation, 2012). When a woman submits a request for one-time financial assistance, it is also not guaranteed that she will be able to secure it. Another option is permanent financial assistance, but this compensation is even rarer than one-time financial assistance. Problems with social workers addressing gendered violence as simultaneously complicit and precarious within the state agenda have been problematized from the same data set elsewhere (Kujundžić, 2022).

In theory, victims of gendered abuse involved in criminal proceedings are entitled to financial compensation for harm suffered. This is called a property-right claim. Property-right claim means that the victim is entitled to compensation for damages, which can be material or immaterial (pain or fear suffered), return of property if the victim can prove that she was the owner or a legal holder of the item in question, and the annulment of some legal transactions if they arose due to the criminal offense (for example, if the defendant forcibly induced the victim to conclude a contract). The survivor (or rather the survivor's attorney) can file a property claim in criminal proceedings or in a special civil lawsuit against the defendant. If the request is made during the criminal proceedings, the precondition for its acceptance is that the court finds the defendant guilty. Bilić, a lawyer specializing in human rights who works with several women's organizations,^18^ explained to me that this right to compensation is also guaranteed by the Directive of the European Parliament and Council (2012/29/EU), which establishes minimum standards on the rights, support and protection of victims of crime. She continued to explain the significance of this to her own work practice:

Through the implementation of the Directive, I have taken the position that I will ask all my clients whether they want to file a property-right claim in criminal proceedings and give me the authority to do so in accordance with the Directive. In 100% of cases I was rejected because the courts do not implement the Directive. And we do not prolong the procedure with this property-right request because we conduct expert examinations on some other circumstances anyway, so that the same expert can report on the same circumstances for determining the amount of the property-legal claim. We will see what the appellate court, possibly the Constitutional Court, will say about this issue, I hope we will not have to go that way, but there is also the European Court [for Human Rights]. In this attempt to help an individual victim, you see that the system is not working in some segment where it should, and you try through an appeal or questioning of a human rights violation to make the system work.

Courts can avoid implementing the Directive, especially if no appeal is made to such practice. Bilić's approach highlights the limitations of the legal advocacy and legalist approach to justice. Progressive laws on victims' protection from gendered violence in practice do not create positive change if they remain positioned within the framework of capitalist socio-economic relations and institutionalized patriarchy (Burcar, 2020). Legislation needs to go beyond a punitive focus, instead prioritizing the prevention of the conditions that enable the continuation of gendered violence. Other hidden costs of getting involved with the legal system in cases of gendered violence are incurred by the proceedings' impact on the victim's everyday life, such as taking time off from work to attend court dates or arranging childcare. Also, if the abusive partner is the sole provider, his imprisonment in custody might mean increased food insecurity and trouble paying rent, mortgage or utility bills, resulting in dependence on welfare benefits and state support.

The number of shelters in Croatia is still insufficient to host thousands of women seeking their services. Shelters are often located in urban centers, making them inaccessible to women without transportation and other resources. The Council of Europe recommends having a women's shelter in every region, one per 10,000 inhabitants, and one social work center for victims of sexual violence per 200,000 women (Mamula and Soba, 2010). Article 23 of the Istanbul Convention states that specialized shelters should be available in each region. President of the Autonomous Women's House Zagreb, Neva Tölle, disputed the Ministry of Social Welfare's claim that there are 19 women's shelters in Croatia. Tölle explained that there are eight shelters for women and children, and the remaining eleven are care homes that receive women and children, amongst other service users (Index Vijesti, 2020). She also indicated that systematic financing of shelters remains a pressing issue.

Women who leave violent relationships often struggle financially, which is exemplified in the time-limited housing for 6–12 months provided by women's shelters. Novak, a shelter worker in a women's shelter founded by the City of Zagreb,^19^ explained how this time is often not enough to secure independence for victims:

All safe houses in Croatia have a limit of one year, therefore by the law, it is temporary accommodation, even the law says from six months to a year in exceptional cases. That sounds awful and is awful sometimes, but the work is being done in the meantime to make the victim independent. To reconnect her with her primary family, to seek help from everyone where help can be sought, to get a job, for children to enrol in kindergarten, school, so that someone can work and earn. It doesn't always end fabulously; it happens to some that they have nowhere to go after that one year, then they can ask to be transferred to another safe house and get more time until they secure independence. Some therefore decide to return to their partner, but most do not return to their partner but return to their primary family. In fact, financial insecurity is just as difficult a problem as the threat of abuse from the perpetrator. Most of the victims are unemployed and without secure housing. Some women have only finished primary or secondary school, making it difficult to find a job, or they find work in the trade sector where all salaries are around 3000 HRK,^20^ so now you need to rent an apartment, buy food, pay for electricity and you have two or three children to care for. This can often be an impossible mission. All this can be solved, but it takes time, for example, to collect alimony from the perpetrator of violence for the children, and to find a decent job.

It is evident that shelters try to mitigate the financial problems of abused women, but often struggle to provide adequate help due to this legal time constraint. The city-founded shelter in which Novak works also only accepts women who have reported the abuse to the police, or who have been referred to the shelter by the social welfare center after reporting abuse to the police. This leaves one fewer option for women who are unable or unwilling to report their partner. This example illustrates how the “caring professions”, such as shelter workers, can still perform policing functions designed to discipline the working class (Munro, 2021).

## 6 “She doesn't realize that she has a barrel of gunpowder next to her”—The legacy of the 1990s war on gendered violence

War veterans hold a special status in the Croatian socio-legal context. They symbolize the sacrifice and righteousness of the “defense” of Croatia in the 1990s. Since then, they have also been organizing into influential organizations and operating as pivotal political actors. Their importance is exemplified by the existence of a separate Ministry of War Veterans founded in 1997, dedicated entirely to their affairs as an integrated system of care for different categories of war veterans. The late President of the Supreme Court, Milan Vuković, remains famous in the Croatian legal and political imaginary as the legal expert who declared it impossible to commit a crime during the liberating “Homeland War” in the 1990s (Šimićević, 2021). This statement illustrates the issues with wartime crime impunity (Ponoš, 2020) as well as the Croatian veterans' revolt against Croatia's cooperation with the Hague Tribunal (Dolenec, 2017).

Related to gendered violence, the status of a war veteran is often used by the courts as a mitigating circumstance. Judge Krznarić remarked upon this point^21^ recalling a case of domestic abuse in which the perpetrator was a war veteran:

The eldest son testified against his father, retelling how his dad beats his mom, beats them [the children], and how he doesn't give them any money. The hardest thing for him to tell us was hearing his mum yelling, Please don't do this anymore,” and all of them [the children] hearing that and knowing what he is doing to her. After all of that, the Supreme Court reduced his sentences by three or four years, and the mitigating circumstance was that he was a war veteran. But he was obviously mentally unwell! And when you think about it, who are those who volunteer to go to war? Some of them already had issues with aggression, getting into fights and violence.

Croatia underwent a significant mobilization during the 1990s war. Exact numbers of people who took part in the war are difficult to pinpoint, but it is estimated that around 11.7% of the Croatian population are war veterans (Dolenec, 2017). In comparison, the United States has approximately 2.9% of its population as veterans of war (Dolenec, 2017). Furthermore, the Croatian Armed Forces, formed through the widespread participation in the 1990s war, emerged as a key institution of the modern Croatian state, affecting both the administrative state structure and the broader societal framework. This is evident in the existence of a wide range of advantages in social policy, in cash and in-kind, for war veterans, their families and survivors (Stubbs and Lendvai-Bainton, 2020). These rights are to a great extent passive and passed on generationally, with the offspring of war veterans being entitled to positive discrimination regarding higher education enrolment (Stubbs and Lendvai-Bainton, 2020). These benefits constitute around 2% of GDP for war veterans. In comparison, social assistance for the poor constitutes only 0.4% of GDP (Stubbs and Lendvai-Bainton, 2020), which shows the prevalence of the state favoring war veterans at the expense of other socially vulnerable groups. Dolenec (2017) argued that war veterans constitute a privileged group that receives substantial compensation from the government, yet they are not seen as a burden to the state. In contrast, initiatives aimed at women and children, often viewed as “dependents” in society, typically consist of mere expressions of concern that lack sufficient funding (Dolenec, 2017, p. 72).

Spouses of war veterans suffering from PTSD are often exposed to emotional, verbal and physical abuse, facing the burden of responsibility and accountability for the whole family (Zdjelarević et al., 2011). This point was elaborated on by a former minister of social welfare, Bajović^22^ who was a member of HDZ known for her Catholic devotion. Her reflection on the issue of war veterans and domestic violence was based on situations she observed as a social worker. Bajović remarked that we still have a lot of people who were war veterans, and the older they get, the weaker their nerves become, “and he needs one wrong word, and later he is sorry, but he can really hurt you.”

She elaborated on what she sees as a problem and a solution to war veterans' abuse issues:

Often the woman doesn't know when to stop, she doesn't realise that she has a barrel of gunpowder next to her, nobody told her that, and instead of working with her and telling her to, look, when he is like that, don't provoke him, in the end you chose him, and the older he gets the worse it will be and you have to be prepared to be tolerant. If he's okay as long as you don't oppose him, if he's bearable, then don't provoke when you can't listen to him, just go away, and if it's really unbearable, then move away, break up, don't create a bad situation. I've seen a lot of cases where a woman starts [provoking] and then she doesn't stop, and I would tell her: ‘Don't you hear yourself; can't you see he will blow up?!' Because I could see that he was refraining himself because there are strangers present otherwise, he would strike her. And if there is no one around, if that is the pattern of behaviour, of course, he will hit her. And if a child sees it, and it has happened a few times, there you have domestic violence. And actually, when you look at it, it was provoked by nothing, because someone has a problem they didn't deal with, didn't treat, just a man with a short fuse and he just cracks.

As mentioned above, war veterans hold a powerful position in Croatian society: they are organized in influential organizations, they influence and are influenced by HDZ, and they are revered as the nation's heroes (Čepo, 2020). At the same time, the spouses of war veterans suffering from PTSD remain affected by not only secondary trauma but also abuse (Zdjelarević et al., 2011; Peraica et al., 2014). The privileging of war veteran status at the expense of victims of gendered violence signals Croatia's condoning of violence when it serves the political and nationalistic agenda. Furthermore, the influence of militarization, the protected status of war veterans and impunity for GBV has further implications for the broader field of post-conflict GBV studies beyond the national level. Although there is a considerable body of literature addressing wartime sexual violence (Zarkov, 2001; Skjelsbaek, 2010; Helms, 2013; Henry, 2014), it is essential to understand gendered violence as a continuum, especially in the light of the increasing militarization occurring in Europe and elsewhere. In Bosnia and Herzegovina, this approach is proposed by Kostovicova et al. (2020), who argued that post-conflict GBV is rooted in the cumulative political and socio-economic dynamics of war and peace, highlighting how war and post-war economic processes co-constitute enduring material conditions that sustain such violence. By framing gendered violence as a continuum sustained by nationalistic and militarized logics, this framework offers a critical lens for examining similar patterns of impunity and structural violence in other post-conflict societies where militarism and war veterans are celebrated and prioritized over gendered justice.

Mandatory military service was abolished in Croatia in 2007; however, plans are in place to reintroduce it starting in the fall of 2025. Since 2016, Croatia has increased its investments in the army by 112% (Hina, 2025) and additional investments in mandatory military service are planned alongside the increased allocation for defense to five per cent of GDP to match other NATO country members. The increased militarization will only add to the glorification and normalization of violence in everyday life, including gendered violence. The priorities of Croatia in joining the war mongering of the EU show a sad departure from the once non-aligned internationalist politics of Yugoslavia. It is expected that increased spending on the military will lead to higher taxation and increased state debt, affecting those already marginalized by the growth-oriented economy. For the survivors of gendered abuse, it means more symbolic lip service and a lack of actual material care and investment.

## 7 Conclusion

This paper focused on the role of redistribution in relation to gendered violence in post-conflict, post-socialist Croatia. It explored the long-term consequences of the transition and post-war periods, particularly the economic and political restructuring that followed the dissolution of Yugoslavia. These processes were marked by poverty, unemployment and the devastation of the social networks entwined with a nationalist agenda that favored traditional gender norms, further reinforced with the growing influence of the Catholic Church.

Empirically, the paper has demonstrated that while the ideal of a working woman still prevails, her material position has fundamentally changed. The proliferation of insecure, low-paid, part-time employment, coupled with the weakening of the welfare support, has left many of the survivors economically dependent on their abusers. These findings challenge the dominant framings of GBV that fail to account for how neoliberal economic policies and post-conflict nationalistic projects create structural conditions that reproduce and legitimize violence. For example, the special status granted to war veterans, some of whom are perpetrators of domestic abuse, not only shields them from accountability but also enshrines a form of gendered impunity which aligns state violence with patriarchal power. This not only has devastating consequences for survivors of gendered violence and abuse but also on society as a whole, since the message is sent to future generations that wartime participation is a glorified excuse for violence. This message is further enshrined in the reintroduction of mandatory military service, coupled with the EU's and NATO's warmongering efforts, which mandate increased military spending for the member states across the board. The system rooted in violence cannot be expected to combat gendered violence seriously.

The paper also intervened in the broader GBV literature, which often centers on recognition politics, such as legal rights, institutional responsiveness, and EU-aligned reforms, while neglecting the material conditions that shape both gendered vulnerability and the limits of institutional protection. By foregrounding redistribution and situating gendered violence within the broader framework of capitalist transition, this paper extends Marxist-feminist theory. It contributes an empirically grounded perspective to feminist analysis of post-conflict contexts. By demonstrating how the erosion of the commons, privatization of the public goods, and defunding of social provisions have deepened women's material precarity, it has made clear how the survivors' ability to leave abusive relationships or seek justice has been structurally undermined.

Theoretically, the analysis presented in this paper pushes GBV scholarship to move beyond institutional and carceral logics, instead recognizing that gendered violence is sustained by the very social and economic systems purported to protect the survivors of abuse. It offers a materialist critique of state complicity in violence and underscores that any feminist praxis must center redistribution, care, and dismantling of militarized, exploitative structures. In doing so, the paper also advanced a materialist and abolitionist approach in the post-conflict context, one that insists that gendered violence cannot be addressed meaningfully without confronting the broader political economy of exploitation, austerity and nationalism. In other words, without redistributive justice, gender justice remains unattainable. Addressing gendered violence requires more than policy reform; it requires a fundamental transformation of the social relations that produce and sustain it.

## Data Availability

The datasets presented in this article are not readily available because of confidentiality issues. Requests to access the datasets should be directed to the corresponding author.

## Footnotes

^1^Known as the “Fimi media” affair, in which HDZ obtained illegal funds, concealed realized incomes and used them to finance the work of a political party from 2004 until 2009. The Prime Minister of that time Ivo Sanader has also been convicted as a war profiteer. For context see (Vuletic 2014).

^2^In the literature there are debates between Marxist-Feminism, Socialist Feminism and Materialist Feminism which go beyond the scope of this article. For further discussion see Čakardić (2022), Fakier et al. (2021), Gimenez (2001), Arruzza (2016), Luxton (2014).

^3^During the Kingdom of Yugoslavia, marriage was under the jurisdiction of religious communities: Catholics, Orthodox, Muslims and other communities all had their own rules. Only a small part of the Kingdom of Yugoslavia was subject to the Hungarian Civil Code. Divorce was very difficult to obtain and depended on the religious community, and marriage rights and obligations were different everywhere. For more info see Simic (2018).

^4^See (Bonfiglioli 2021).

^5^For a critique of the concept of transition see Buden (2010).

^6^There were some who profiteered of the wartime and transition period, either by war profiteering or by buying and reselling what was once state property. See Čučković (1993), Čengić (1996), Kostovicova et al. (2020).

^7^This is the term used in the official Croatian nationalist narrative (in some cases also “Greater Serbian Aggression on Croatia”) to denote the 1990s war of dissolution of Yugoslavia.

^8^*Ministarstvo branitelja*, literal translation should read “Ministry of Defenders.”

^9^Interview conducted in Zagreb on 10.9.2019.

^10^Interview conducted in Zagreb on 19.7.2019.

^11^Interview conducted in Zagreb on 1.9.2018.

^12^Approximately 229.83 and 344.74 GBP.

^13^Approximately 57.46 GBP.

^14^Approximately 206.84 GBP.

^15^Interview conducted in Zagreb on 17.7.2018.

^16^Interview conducted in Zagreb on 25.2.2019.

^17^Interview conducted in Zagreb on 17.7.2019.

^18^Interview conducted on 6.9.2019.

^19^Interview conducted in Zagreb on 10.7.2018.

^20^Approximately 344.74 GBP.

^21^Interview conducted in Zagreb on 7.8.2018.

^22^Interview conducted in Zagreb on 30.4.2019.

## References

[B1] Ajduković M. Pavleković G. (2003). Nasilje nad ženom u obitelji. Zagreb: Društvo za psihološku pomoć.

[B2] Arruzza C. (2016). Functionalist, determinist, reductionist: social reproduction feminism and its critics. Sci. Soc. 80, 9–30. 10.1521/siso.2016.80.1.9

[B3] Arruzza C. Gawel K. (2020). The politics of social reproduction. An introduction. CLCWeb Comp. Lit. Cult. 22, 1–7. 10.7771/1481-4374.383629356800

[B4] Arsel Z. (2017). Asking questions with reflexive focus: a tutorial on designing and conducting interviews. J. Consum. Res. 44, 939–948. 10.1093/jcr/ucx096

[B5] Bežovan G. (2003). Utjecaj organizacija civilnog društva u Hrvatskoj (The influence of civil society organizations in Croatia). Rev. Sociol. 2, 127–142. Croatian. Available online at: https://hrcak.srce.hr/14495

[B6] Bhattacharya T. (2013). Explaining gender violence in the neoliberal era. Int. Soc. Rev. 91, 2013–2014. Available online at: https://isreview.org/issue/91/explaining-gender-violence-neoliberal-era/

[B7] Bhattacharya T. (2017). Social Reproduction Theory: Remapping Class, Recentering Oppression. London: Pluto Press.

[B8] Bonfiglioli C. (2019). Women and Industry in the Balkans: The Rise and Fall of the Yugoslav Textile Sector. London: Bloomsbury Publishing.

[B9] Bonfiglioli C. (2020). Post-socialist deindustrialisation and its gendered structure of feeling: the devaluation of women's work in the Croatian garment industry. Labor Hist. 61, 36–47. 10.1080/0023656X.2019.1681643

[B10] Bonfiglioli C. (2021). Women's internationalism and Yugoslav-Indian connections: from the non-aligned movement to the UN decade for women. Natl. Pap. 49, 446–461. 10.1017/nps.2020.11

[B11] Boric R. (2003). Feminizam i etika- kriticke analize. Zarez- dvotjednik za kulturna i drustvena zbivanja (Feminism and Ethics—Critical Analyses. Zarem—Biweekly Magazine for Cultural and Social Events), 2–47.

[B12] Brown W. (1995). States of Injury, Power and Freedom in Late Modernity. Princeton, NJ: Princeton University Press.

[B13] Buden B. (2010). Children of postcommunism. Radical Philos. 9, 18–25. Available online at: https://www.radicalphilosophy.com/article/children-of-postcommunism

[B14] Burcar L. (2020). Restauracija kapitalizma: repatrijarhalizacija drustva (The Restoration of Capitalism: The Repatriarchalization of Society). Zagreb: Institut za etnologiju i folkloristiku, Centar za zenske studije.

[B15] Čakardić A. (2022). Who cares? Neoliberalism, informal labour, and life-making. Sociologija LXIV, 504–518. 10.2298/SOC2204503C

[B16] Čengić D. (1996). Privatisation and management buy-out: The example of Croatia. Commun. Econ. Econ. Transform. 8, 549–563. 10.1080/14631379608427871

[B17] Čepo D. (2020). “Structural weaknesses and the role of the dominant political party: democratic backsliding in Croatia since EU accession,” in Southeast European and Black Sea Studies (London: Routledge), 2–19.

[B18] Combahee River Collective (2017). How We Get Free: Black Feminism and the Combahee River Collective, ed. K.-Y. Taylor (Chicago: Haymarket Books).

[B19] Cowan L. (2024). Why Would Feminists Trust the Police? London: Verso.

[B20] Čučković N. (1993). Privatisation in Croatia: what went wrong? Hist. Eur. Ideas 17, 725–733. 10.1016/0191-6599(93)90095-8

[B21] Dolenec D. (2017). A soldier's state? Veterans and the welfare regime in Croatia. Anali Hrvatskog Politoloskog Drustva (Ann. Croat. Polit. Sci. Soc.) 14, 55–77. Croatian. 10.20901/an.14.03

[B22] Drucker P. (2015). Warped: Gay Normality and Queer Anti-Capitalism, Historical Materialism Book Series. Leiden: Brill.

[B23] Duff K. (2017). The criminal is political: policing politics in real existing liberalism. J. Am. Philos. Assoc. 3, 485–502. 10.1017/apa.2017.39

[B24] Duff K. (2018). Feminism against crime control: on sexual subordination and state apologism. Hist. Mater. 26, 123–148. 10.1163/1569206X-00001649

[B25] Duff K. Jenkins K. (2021). “*More Policing Won't Stop Gendered Violence”*. London: Verso Books. Available online at: https://www.versobooks.com/blogs/news/5094-more-policing-won-t-stop-gendered-violence (Accessed September 1, 2025).

[B26] Elias J. Rai S. M. (2019). Feminist everyday political economy: space, time, and violence. Rev. Int. Stud. 45, 201–220. 10.1017/S0260210518000323

[B27] Ellsberg M. Carroll L. Heise L. (2005). Researching Violence Against Women: A Practical Guide for Researchers and Activists. Washington, DC: World Health Organization

[B28] Fakier K. Mulinari D. Räthzel N. (eds) (2021). Marxist-Feminist Theories and Struggles Today. London: Zed Books.

[B29] Fook J. (1999). Reflexivity as method. Ann. Rev. Health Soc. Sci. 9, 11–20. 10.5172/hesr.1999.9.1.11

[B30] Fraser N. (2013). Fortunes of Feminism: From State-Managed Capitalism to Neoliberal Crisis. London: Verso Books.

[B31] Galić B. (2018). A case study of retraditionalization and clericalization of the croatian society: “feminist threat” at the governing position of a higer education institution. Casopis Sociologija (J. Sociol.) 44972, 272–497. Slovenian. 10.2298/SOC1801210G

[B32] Ghodsee K. (2018). Why Women Have Better Sex Under Socialism. London: Penguin Random House UK.

[B33] Gimenez M. E. (2001). Marxism, and class, gender, and race: rethinking the trilogy. Race Gender Class 8, 23–33. Available online at: https://www.jstor.org/stable/41674970

[B34] Goodmark L. (2018). Decriminalizing domestic violence. Oakland, CA: University of California Press.

[B35] Goodmark L. (2023). Imperfect Victims: Criminalised Survivors and The Promise of Abolition Feminism. Oakland, CA: University of California Press.

[B36] Gudac Dodic V. (2006). Zena u socijalizmu. Belgrade: INIS.

[B37] Gužvica S. Bilić D. (2024). Zavisna Država Hrvatska. Zagreb: Bilten. Available online at: https://www.bilten.org/?p=47506 (Accessed September 1, 2025).

[B38] Haug H. K. (2012). Creating a Socialist Yugoslavia: Tito, Communist Leadership and the National Question. London: I.B. Tauris.

[B39] Helms E. (2013). Innocence and Victimhood: Gender, nation, and women's activism in postwar Bosnia-Herzegovina. Madison, WI: University of Wisconsin Press.

[B40] Hemmings C. (2012). Affective solidarity: feminist reflexivity and political transformation. Fem. Theory 13, 147–161. 10.1177/1464700112442643

[B41] Henry N. (2014). The fixation on wartime rape: feminist critique and international criminal law. Soc. Legal Stud. 23, 93–111. 10.1177/0964663913499061

[B42] Hina (2025). Jandroković: Izdvajanje pet posto za obranu nužno zbog sigurnosne situacije. Zagreb: Ius-Info. Available online at: https://www.iusinfo.hr/aktualno/dnevne-novosti/jandrokovic-izdvajanje-pet-posto-za-obranu-nuzno-zbog-sigurnosne-situacije-65353 (Accessed September 1, 2025).

[B43] Index Vijesti. (2020). Neva Tölle: Država kaže da ima 19 skloništa za žene. Nema, samo ih je osam – Index.hr, Index.hr, 22 September. Available online at: https://www.index.hr/vijesti/clanak/neva-t?lle-drzava-kaze-da-ima-19-sklonista-za-zene-nema-samo-ih-je-osam/2216078.aspx (Accessed April 19, 2021).

[B44] Jansen S. (2005). Antinacionalizam: etnografija otpora u Beogradu i Zagrebu [Antinationalism: Ethnography of Resistance in Belgrade and Zagreb]. Belgrade: Biblioteka XX Vek.

[B45] Jovanovic A. (2015). Antifascist Front of Women within the socialist transformation of society, Udruženje za kulturu i umjetnost Crvena. Available online at: http://afzarhiv.org/items/show/443

[B46] Kamenov Z. Galic B. (2011). Rodna ravnopravnost i diskriminacija u Hrvatskoj. Biblioteka ONA Ured za ravnopravnost spolova vlade Republike Hrvatske. Available online at: https://ravnopravnost.gov.hr/UserDocsImages/arhiva/images/pdf/dokumenti/Istra%C5%BEivanje%20Rodna%20ravnopravnost%20i%20diskriminacija%20u%20Hrvatskoj_%20Percepcija,%20iskustva%20i%20stavovi%20o%20rodnoj%20diskriminaciji%20u%20Republici%20Hrvatskoj.pdf

[B47] Kandal R. T. (1995). Gender, Race and Ethnicity: Let's Not Forget Class. Race Gender Class J. 2, 139–162.

[B48] Kesic V. (1994). A response to Catharine MacKinnon's article “Turning rape into pornography: postmodern genocide”. Hastings Women's Law Journal, 5, 267–280.

[B49] Kocman J. (1978). Ž*ene Jugoslavije u radničkom pokretu i ženskim organizacijama 1918-1941*. Belgrade: Narodna Knjiga.

[B50] Korolczuk E. Graff A. (2018). Gender as “Ebola from Brussels”: The Anticolonial Frame and the Rise of Illiberal Populism. Signs J. Women Cult. Soc. 43, 797–821. 10.1086/696691

[B51] Kostovicova D. Bojicic-Dzelilovic V. Henry M. (2020). Drawing on the continuum: a war and post-war political economy of gender-based violence in Bosnia and Herzegovina. Int. Fem. J. Politics 22, 250–272. 10.1080/14616742.2019.1692686

[B52] Kovats E. (ed.). (2017). The Future of the EU: Feminist perspectives from East-Central Europe. Budapest: Friedrich-Ebert-Stiftung Budapest.

[B53] Kujundžić J. (2022). Social work and domestic violence in croatia through a gendered lens: between power and precarity. Revija za Sociologiju (J. Sociol.) 52, 331–358. Crotian. 10.5613/rzs.52.3.3

[B54] Kvale S. Brinkmann S. (2009). InterViews: Learning the Craft of Qualitative Research Interviewing. London and Thousand Oaks: SAGE Publications.

[B55] Lewis H. (2016). The Politics of Everybody. London: Zed Books.

[B56] Lóránd Z. (2019). “Feminism and violence against women in Yugoslavia during state socialism,” in Women, Global Protest Movements, and Political Agency: Rethinking the Legacy of 1968, eds. S. Colvin and K. Karcher (London: Routledge), 84–97.

[B57] Lottholz P. (2024). “Illiberalism of their own making?: A post-liberal critique of illiberalism research,” in The Oxford Handbook of Illiberalism, ed. M. Laruelle (Oxford: Oxford University Press), 155–174.

[B58] Luxton M. (2014). Marxist feminism and anticapitalism: reclaiming our history, reanimating our politics. Stud. Polit. Econ. 94, 137–160. 10.1080/19187033.2014.11674957

[B59] Mamula M. Soba Ž. (2010). Organizacije civilnog društva koje pružaju specijalizirane servise ženama žrtvama nasilja kao ključni akteri u procesu demokratizacije društva. Ženska soba – Centar za seksualna prava. Available online at: https://www.zenskasoba.hr/docs/OCD_i_kljucni_akteri.pdf

[B60] Maslić Seršić D. (2010). Ekonomsko nasilje nad ženama: manifestacije, posljedice i putovi oporavka. Zagreb: Autonomna ženska kuća Zagreb, Ministarstvo obitelji, branitelja i medugenercijske solidarnosti.

[B61] Miller S. L. (2001). The paradox of women arrested for domestic violence: criminal justice professionals and service providers respond. Violence Against Women 7, 1339–1376. 10.1177/10778010122183900

[B62] Miškovska Kajevska A. (2017). Feminist Activism at War: Belgrade and Zagreb feminists in the 1990s. London: Routledge.

[B63] Munro K. (2021). Unproductive Workers and State Repression. Rev. Radic. Polit. Econ. 53, 623–630. 10.1177/0486613421104328419465768

[B64] Nacional (2018). Ministarstvo branitelja odlučivati će o statusu žrtve silovane u zatvoru Kuline (Ministry of War Veterans will decide on the status of the victim raped in the Kuline prison). Zagreb: Nacional.hr. Available online at: https://www.nacional.hr/ministarstvo-branitelja-odlucivati-ce-o-statusu-zrtve-silovane-u-zatvoru-kuline/ (Accessed August 31, 2025).

[B65] Naeem M. Ozuem W. Howell K. Ranfagni S. (2023). A step-by-step process of thematic analysis to develop a conceptual model in qualitative research. Int. J. Qual. Methods 22, 1–18. 10.1177/16094069231205789

[B66] Neumann P. (2017). When laws are not enough: Violence against women and bureaucratic practice in Nicaragua. Soc. Forces 95, 1105–1125. 10.31235/osf.io/6gw4c33784477

[B67] Nikolić-Ristanović V. (2002). Social Change, Gender and Violence: Post-Communist and War Affected Societies. Berlin: Springer. 10.1007/978-94-015-9872-9

[B68] Nikolić-Ristanović V. Dokmanović M. (2006). International Standards on Domestic Violence and Their Implementation in the Western Balkans. Belgrade: Prometej.

[B69] Noyé S. Rebucini G. (2021). “Queer as Materialism”, in *Oxford Research Encyclopedia of Politics*. Oxford: Oxford University Press.

[B70] Olick J. K. Stiglmayer A. (1995). Mass rape: the war against women in bosnia-herzegovina. Contemp. Sociol. 24:332. 10.2307/2076482

[B71] Olufemi L. (2020). Feminism, Interrupted. London: Pluto Press.

[B72] Patton M. Q. (2015). Qualitative Research and Evaluation Methods, 4th edn. London and Thousand Oaks: SAGE Publications.

[B73] Peraica T. Vidović A. Petrović Z. K. Kozarić-Kovačić D. (2014). Quality of life of Croatian veterans' wives and veterans with posttraumatic stress disorder. Health Qual. Life Outcomes 12, 1–9. 10.1186/s12955-014-0136-x 25209006 PMC4172835

[B74] Ponoš T. (2020). Sisak 1990-1991: ratni zločini nad Srbima. Tragovi: časopis za srpske i hrvatske teme (Tragovi Mag. Serbian Croatian Topics) 3, 7–72. Crotian. Available online at: https://hrcak.srce.hr/246543

[B75] Postmus J. L. Hoge G. L. Breckenridge J. Sharp-Jeffs N. Chung D. (2020). Economic abuse as an invisible form of domestic violence: a multicountry review. Trauma Violence Abuse 21, 261–283. 10.1177/1524838018764160 29587598

[B76] Radačić I. (2014). Seksualno nasilje: Mitovi, stereotipi i pravni sustav. Zagreb: Tim Press.

[B77] Rajter M. (2015). Basline Study to Map Child Protection Practices and Related Workforce Needs in Southeast Europe CROATIA Final Report. Zagreb: Child Protection Hub for South East Europe.

[B78] Ramet S. (Ed.). (1999). Gender Politics in the Western Balkans: Women and Society in Yugoslavia and the Yugoslav Successor States, ed. S. P. Ramet. University Park, PA: The Pennsylvania State University.

[B79] Rodriguez Martinez P. (2011). Feminism and violence: the hegemonic second wave's encounter with rape and domestic abuse in USA (1970–1985). Cult. Dyn. 23, 147–172. 10.1177/0921374011430566

[B80] Ryan-Flood R. Gill R. (2010). Secrecy and Silence in the Research Process: Feminist Reflections. London: Routledge.

[B81] Saldaña J. (2016). The Coding Manual for Qualitative Researchers. London and Thousand Oaks: SAGE Publications.

[B82] Sarkar A. (2025). Minority Rule. London: Bloomsbury Publishing.

[B83] Sarnavka S. Markulin Ž. (Eds.). (2013). Što znamo o ekonomskom nasilju nad Ženama? BaBe!

[B84] Simic I. (2018). Soviet Influences on Postwar Yugoslav Gender Policies. London: Palgrave Macmillan.

[B85] Šimićević H. (2021). Poslijeratni zločinci. Portal Novosti. Available at: https://www.portalnovosti.com/poslijeratni-zlocinci/ (Accessed September 24, 2021).

[B86] Šipuš V. (2023). Uloga Programa “Zaželi- Program Zapošljavanja Žena” Kao Oblika Deinstitucionalizirane Skrbi. Marsonia: Casopis za društvena i humanistička istraživanja (Marsonia J. Soc. Human. Res.) 2, 61–74. Crotian. Available online at: https://hrcak.srce.hr/file/453595

[B87] Skjelsbaek I. (2010). The Elephant in the Room: An Overview of How Sexual Violence came to be Seen as a Weapon of War. Oslo: Peace Research Institute Oslo (PRIO).

[B88] Sørensen M. J. Wiksell K. (2019). Constructive resistance to the dominant capitalist temporality. Sociologisk Forskning (Sociol. Res.) 56, 253–274. Danish. 10.37062/sf.56.18802

[B89] Stubbs P. Lendvai-Bainton N. (2020). Authoritarian Neoliberalism, Radical Conservatism and Social Policy within the European Union: Croatia, Hungary and Poland. Dev. Change 51, 540–560. 10.1111/dech.12565

[B90] Stubbs P. Sertić N. (1996). Nevladine organizacije, ulaganje sredstava i socijalno blagostanje: neke hipoteze o Hrvatskoj i Sloveniji. Revija za socijalnu politiku (Soc. Policy Rev.), 3, 25–30. Croatian. 10.3935/rsp.v3i1.489

[B91] Stubbs P. Žitko M. (2024). Beyond crony capitalism: financialization, flexible actors and private power in transition economies—the case of Agrokor. Socio-Econ. Rev. 22, 811–834. 10.1093/ser/mwad035

[B92] Stylianou A. M. (2018). Economic abuse within intimate partner violence: a review of the literature. Viol. Vict. 33, 3–22. 10.1891/0886-6708.33.1.3 29195516

[B93] Táíwò O. O. (2022). Elite Capture: How the Powerful Took Over Identity Politics (And Everything Else). London: Pluto Press.

[B94] The Advocates for Human Rights Autonomna Ženska Kuća Zagreb and Bulgarian Gender Research Foundation. (2012). Implementacija hrvatskog zakonodavstva vezanog uz partnersko nasilje. Zagreb: Autonomna Ženska Kuća Zagreb.

[B95] UNICEF (1994). Women and Gender in Countries in Transition: A UNICEF Perspective. New York: Regional Office for Central and Eastern Europe.

[B96] Vuletic I. (2014). “The Organised Structure of Power” and Economic Crime “FIMI-Media” Case and a View from the Croatian Perspective. J. Law Crim. Just. 2, 133–149. 10.15640/jlcj.v2n2a8

[B97] Zarkov D. (2001). “The body of the other man: sexual violence and the construction of masculinity, sexuality in Croatian media,” in Victims, Perpetrators or Actors? Gender, Armed Conflict and Political Violence, ed. C. O. N. Moser and F. Clar (London: Zed Books), 69–81.

[B98] Žarkov D. (2003). Feminism and the disintegration of Yugoslavia: on the politics of gender and ethnicity. Soc. Dev. Issues 24, 1–19. Available online at: https://repub.eur.nl/pub/23361

[B99] Žarkov D. (2007). The Body of War. Durham: Duke University Press.

[B100] Žarkov D. Drezgić R. (2006). Feministicke Nevolje s Balkanom: feminist troubles with the Balkans. Sociologija, XLVII, 290–306. 10.2298/SOC0504289D

[B101] Zaviršek D. (2008). Engendering social work education under state socialism in Yugoslavia. Br J Soc Work 38, 734–750. 10.1093/bjsw/bcn022

[B102] Zdjelarević A. Komar Z. Lončar M. Dijanić Plašć I. Hrabač P. Groznica I.. (2011). Quality of life in families of Croatian veterans 15 years after the war. Coll. Antropol. 35, 281–286. Available online at: https://hrcak.srce.hr/64093 21648348

